# Incidence, Management, and Outcome of Molar Pregnancies at a Tertiary Care Hospital in Quetta, Pakistan

**DOI:** 10.5402/2011/925316

**Published:** 2011-10-16

**Authors:** Mahrukh Fatima, Pashtoon Murtaza Kasi, Shahnaz Naseer Baloch, Masoom Kassi, Shah Muhammad Marri, Mahwash Kassi

**Affiliations:** Department of Obstetrics and Gynecology, Bolan Medical College, 8-13/36 Kasi Road, Quetta, Balochistan 87300, Pakistan

## Abstract

Molar pregnancies represent a significant burden of disease on the spectrum of gestational trophoblastic diseases. The incidence appears to be higher in women from South Asia. The purpose of our prospective study was to determine the incidence, presentation, and outcomes of all molar pregnancies at our institution. During the study period, there were a total of 16,625 patients admitted to our department; out of whom 85 patients were diagnosed with a molar pregnancy. Vaginal bleeding was the commonest symptom (94.2%); theca lutein cysts were noted in 39% of the cases. Suction, dilatation, and curettage were noted to be the preferred method in almost all cases; hysterectomy was done in 12 (14.1%) patients. Single-agent chemotherapy was employed in high-risk patients and was well tolerated. Mean followup for these patients was 5.7 months (range 1–24 months). None of these patients developed persistent trophoblastic disease, invasive mole, or choriocarcinoma during the follow-up period.

## 1. Introduction

Molar pregnancies represent a significant burden of disease on the spectrum of gestational trophoblastic diseases. The incidence appears to be higher in women from South Asia, including a trend towards recurrent molar pregnancies [1, 2]. This higher trend in some populations has been attributed to “nutritional and socioeconomic status” [3]. 

The purpose of our study was to review all the molar pregnancies at our institution. Our specific aims were then to determine the incidence, the associated morbidity, presentation, risk factors, and complications noted at our institution. This data would really be helpful in the context of the city which serves as the tertiary referral center for all the cases from the largest province of the country of Pakistan.

## 2. Materials and Methods

Ethical approval for the study was obtained from the Department of Obstetrics and Gynaecology, Bolan Medical College, Quetta, Pakistan, as well as the local ethical committee for research, and the research conducted was performed according to the Declaration of Helsinki. 

The study was a prospective study carried out at the largest tertiary care government hospital in the city of Quetta in Balochistan, the largest province in Pakistan. Quetta is a metropolitan city and the capital of the province. People belonging to different castes live here along with many refugees who were from the adjacent war-torn country of Afghanistan and migrated during the early 1980s and 1990s. This represents one of the major teaching/tertiary care centers for the province. 

Due to lack of computerized medical records, we started our study from 1994 and were able to collect data for all cases that needed EPH in Gynae units I and II at Sandeman Medical College Hospital, Quetta, Pakistan, over period of 2 years from September 25th 1994 to September 1st 1996. This work was done as part of thesis for Dr. F. Mahrukh's FCPS degree and now is being sent for publication after completion of her degree. Furthermore, records after transfer of the gynecology department to another institution were not available for review. 

During this period, there were a total of 16,625 patients admitted to the both units of obstetrics and gynecology at our institution. 85 patients were diagnosed and confirmed with histopathological findings to have a molar pregnancy. Following identification of these patients, data regarding their basic demographics, risk factors, associated complications and followup were then collected and entered into a database developed in Microsoft Access 2000. An attempt to follow up all 85 patients was done for at least 2 years from the time of initial surgical evacuation. This data was then imported into the Statistical Package for Social Sciences version 14.0 (SPSS Inc, Chicago, Ill, USA) for further analysis.

## 3. Results and Discussion

As noted above, 85 patients (0.51%) were diagnosed with a molar pregnancy from a total of 16,625 patients admitted to the institution. This translates into an incidence of ~5.1 per 1,000 patients admitted to the institution. 

In majority of the patients, the classical presentation was that of delayed menstrual periods suggestive of pregnancy and vaginal bleeding. When combined with findings of an out of proportion enlargement of the uterus and absent fetal heart tones, the diagnosis of a molar pregnancy was suspected. This was then confirmed by measuring the serum beta-hCG (*β*-hCG) and sonography. All sonograms were performed by author F. Mahrukh herself and were noted to be helpful in aiding the diagnosis in 79.5% of the cases. Whereas, *β*-hCG was noted to be elevated in all patients and was more than 50,000 MIU/mL in 85.8% of the cases. 

Table 1 outlines the incidence of hydatiform mole in relation to parity and gravidity along with other associated factors. 

Although hydatidiform mole is more common in primigravidas, in our study most patients were multigravida. Among 85 cases of HM, the range of parity was from 0 to 17. Gravida above 5 apparently is considered as a poor prognostic sign.

Excessive uterine size is one of the classic signs of HM. As outlined in Table 1, in our study more than 70% of cases had size of uterus 4–12 weeks greater than gestational age; and more than 17% cases had size of uterus more than 12 weeks greater. “Excessive uterine size is usually associated with markedly elevated levels of human chorionic gonadotropin (hCG) from trophoblastic overgrowth” [1]. Likewise, excessive uterine size was noted in 21/74 (28%) of patients at the New England Trophoblastic Center [4]. 

Similarly, theca lutein cysts develop almost exclusively in patients with very high *β*hCG levels which induce ovarian hyper stimulation and produce bilateral multilocular ovarian cysts. In this study, cysts were found in about 39% of cases out of which 17.64% patients had cysts greater than 6 cms in size. They usually produce symptoms like pelvic pressure and discomfort in many patients. In most of the cases, they regress spontaneously within 8 weeks. Depending on how the diagnosis of molar pregnancy was made (clinical versus sonographic), the incidence of theca lutein has been quoted to be between 20 and 46% of patients with molar pregnancy [5–7]. Since the advent and frequent use of ultrasound, larger sizes of theca lutein cysts and larger uterine sizes have become less common [8]. Even though in our subset of patients, the cysts were present in about 39% of the cases, none of them needed emergency surgery for torsion. This is similar to another series from Turkey, where only 1 patient needed emergency surgery for torsion [9]. 

Vaginal bleeding was the commonest symptom (94.2%); apart from amenorrhea which was present in all the cases. This was also noted to be the commonest symptom by a large series by Goldstein where it was present in 97% of their patients; and also in a series from China were it was present in 83.2% of the patients with hydatidiform mole [10, 11]. Other signs/symptoms included hyperemesis and preeclampsia. Likewise, preeclampsia and hyperemesis were reported in 12 to 27 percent and 20 to 26 percent of patients and occurred almost exclusively in those with markedly elevated human chorionic gonadotropin values and excessive uterine size [3]. Hyperthyroidism was noted in 1 of our patients. 5 patients were diagnosed on routine sonographic examination. 

These patients were then followed up to see the management they received and their outcomes. Suction, dilatation, and curettage were noted to be the preferred method of management in 62 (72.9%) of the cases. 12 women underwent an elective hysterectomy as primary therapy for intact hydatidiform mole (HM); 5 of whom also underwent with bilateral salpingo-oophorectomy (BSO) and 4 with unilateral salpingo-oophorectomy. All these patients were noted to be older than 40 and had complete their family planning. In patients who underwent salpingo-oophrectomies, reason was noted to be the large size of associated ovarian cyst on one and/or both sides. 

Both suction and sharp curettage specimens were submitted to the department of pathology for histopathological examination and confirmed the diagnosis in all of our cases. 

Persistence of uterine bleeding as a complication was noted in 73 (85.9%) of the patients; with it persisting in 13 (15.3%) patients for more than 2 weeks. The need for transfusion of packed red blood cells (PRBCs) was noted in all patients; with a mean of 2.58 units of PRBCs (Range 1–6). 

Postmolar trophoblastic disease was diagnosed on the basis of a rise of *β*hCG after the initial plateau or with the detection of metastases. 2 patients in our study period developed postmolar trophoblastic disease after complete molar pregnancy. 

After their initial management, patients were noted to be classified into low risk or high risk group based on Goldstein's Mole Prognosis Scoring system [12] and received prophylactic single-agent chemotherapy with methotrexate if they fell into the high risk group (score of more than 4). “Several investigators have reported that prophylactic chemotherapy at the time of molar evacuation reduces the frequency of postmolar tumor [12, 13]. Kim and colleagues reported in a prospective randomized trial that prophylactic Methotrexate reduced the incidence of postmolar tumor from 47 to 14% in patients with high-risk complete mole [14]. Prophylactic chemotherapy may be particularly beneficial in patients with high-risk complete moles when hormonal followup is either unavailable or unreliable.” [15] In patients at our institution, 70 (82.4%) of the patients fell into high risk group; and given the considerable lack of followup of patients presenting at our institution alongside the fact that a bulk of them are Afghan refugees, single-agent prophylactic chemotherapy with Methotrexate is usually employed at our institution if patients fall in the high risk group. 

Serial measurement of serum *β*hCG levels is used to monitor the behavior of resident trophoblastic tissue after surgical evacuation of hydatidiform mole. Various terms are used in this context. A *plateau* is a pattern where there is neither decrease nor an increase >10% or 50% of serum levels over 3 weeks on the basis of three or four consecutive weekly measurement over 3 weeks. Similarly, a *rise* is defined as increasing levels on the basis of two or more consecutive weekly measurements. *Persistent* is when elevation of BhCG is found after 16 weeks of evacuation. Sharp regression is when levels immediately fall after evacuation; while slow regression is when serum levels have regress slowly to normal within 8 to 9 weeks from uterine evacuation. In our study population as noted in Table 2, most of the patients in the high risk group who received single-agent chemotherapy after surgical evacuation had a sharp regression curve while most of the patients in group B had a slow fall over 6–8 weeks.

Followup of these patients was attempted for a period of at least 2 years from the time of surgical evacuation. Mean followup for these patients was 5.7 months (range 1–24 months). None of these patients developed persistent trophoblastic disease, invasive mole, or choriocarcinoma during the follow-up period. Even though, an attempt was made to follow these patients for 2 years, at government hospitals and due to the fact that a bulk of these patients are refugees, followup is a challenging task. This is also a factor in the Government hospitals in the rest of the country as well, and would remain a continuous ongoing challenge [16].

## 4. Conclusions

- In our study, thus we have identified not only the incidence of molar pregnancies at our institution which serves as the largest tertiary care facility for the entire province of Pakistan and neighboring Afghanistan, but also highlighted the significant morbidity, management strategies, and associated complications.
- Limitation of our study includes limited followup for some of these patients, which as noted would remain a continuous ongoing challenge for the tertiary care hospital, since it serves not only the city but essentially the entire province and neighboring Afghanistan.
- Single-agent chemotherapy is well tolerated in patients classified as high risk. As noted earlier by Goldstein et al., prophylactic chemotherapy may be particularly beneficial in patients with high-risk complete moles when hormonal followup is either unavailable or unreliable. Given our institutional setting and the demographics, we would advocate the prophylactic chemotherapy for the high risk group since followup is not entirely reliable.

## Figures and Tables

**Table 1:** Basic Demographics, incidence of hydatidiform mole, and associated factors.

	Number of cases	%
(1) *Hydatidiform mole *	85	0.51% ~5 per 1,000 patients admitted

(2) *Monthly income (Rupees) *		
<5,000 (~$60)	70	82.4%
5,000–10,000 ($60–120)	14	16.5%
>10,000 ($120)	1	1.1%

(3) *Parity *		
0	31	36.5%
1–4	29	34.1%
5-17	25	29.4%

(4) *Size of luteal cysts *		
Less than 6 cm	18	21.2%
≥6 cm	15	17.6%
Not detected	52	61.2%

(5) *Size of the uterus *		
Corresponding to the gestational age	10	11.7%
4–12 weeks more than the gestational age	60	70.6%
>12 weeks	15	17.7%

(6) *Presenting signs/symptoms *		
Amenorrhea	85	100%
Vaginal Bleeding	80	94.2%
Hyperemesis	8	9.4%
Preeclempsia	10	11.8%

(7) *Time from their last menstrual period (LMP) (weeks) *		
≤8	4	4.7%
9–12	22	25.9%
13–20	51	48.2%
>20	18	21.2%

(8) *Baseline anemia *	58	68.2%

(9) *Hyperthyroidism *	1	1.2%

**Table 2:** Diagnosis, management of HM, and associated complications.

	Number of cases	%
(1) *Diagnostic utility *		
Sonographic findings	73	79.5%
*β*hCG	85	100%
Histopathology	85	100%

(2) *βhCG levels *(MIU/mL)		
<50,000	12	14.1%
50,000–100,000	53	62.4%
>100,000	20	23.5%

(3) *Treatment modality *		
Suction/sharp curettage	62	72.9%
Oxytocin/curettage	10	11.8%
Hysterectomy	12	14.1%
Prostaglandins/curettage	1	1.2%

(4) *Persistence of uterine bleeding after evacuation *		
None	12	14.1%
Up to 1 week	60	70.6%
2–3 weeks	12	14.1%
≥4 weeks	1	1.2%

(5) *Need for blood transfusion *	85	100%

(6) *Fever *	22	25.8%

(7) *Sepsis *	2	2.4%

(8) *Respiratory insufficiency *	1	1.2%

(9) *Postmolar trophoblastic disease *	2	2.4%

(10) *Pattern of *β*hCG regression high risk group *		
Sharp regression	66	94.3%
Slow regression	2	2.9%
Plateau	1	1.4%
Rising	1	1.4%
*Low risk group*		
Sharp regression	3	20%
Slow regression	12	80%
